# Influence of Different Commercial Yeasts on Volatile Fraction of Sparkling Wines

**DOI:** 10.3390/foods10020247

**Published:** 2021-01-26

**Authors:** Valeriu V. Cotea, Mihai Cristian Focea, Camelia Elena Luchian, Lucia Cintia Colibaba, Elena Cristina Scutarașu, Niculaua Marius, Cătălin Ioan Zamfir, Andreea Popîrdă

**Affiliations:** 1 “Ion Ionescu de la Brad” University of Agricultural Sciences and Veterinary Medicine, 3rd M. Sadoveanu Alley, 700490 Iași, Romania; vcotea@uaiasi.ro (V.V.C.); foceamihai1@gmail.com (M.C.F.); cintia.colibaba@uaiasi.ro (L.C.C.); cristina_scutarasu@yahoo.com (E.C.S.); p.andreea08@yahoo.com (A.P.); 2 Research Center of Oenology, Romanian Academy—Iași Division, 9th M. Sadoveanu Alley, 700505 Iași, Romania; niculaua@acadiasi.ro (N.M.); catalin.zamfir@acadiasi.ro (C.I.Z.)

**Keywords:** reverse-osmosis, yeasts, ethanol reduction, sparkling wines, volatile compounds

## Abstract

The occurrence of aroma constituents in sparkling wines, with direct impact on their organoleptic characteristics, is affected by several factors, for example the base-wine particularities, grapes cultivar conditions, inoculated yeasts, the aging stage, and wine-making practices. This study evaluated the influence of different four commercial yeasts (IOC FIZZ™, IOC DIVINE™, LEVULIA CRISTAL™, and IOC 18-2007™) on the volatile composition of experimental sparkling wines. For this, five sparkling wines variants from the Muscat Ottonel grape variety were obtained. The base-wine was obtained through reverse osmosis and had a predetermined alcoholic concentration (10.5% vol.). In order to fulfill the proposed purpose, the experimental sparkling wines were characterized by the physical–chemical parameters (according to International Organization of Vine and Wine methods of analysis), volatile fraction (using gas-chromatography coupled with mass spectrometry technique), and sensory descriptors. Data showed a key impact on the concentration of the volatile constituents (*p* < 0.05), depending on the type of inoculated yeast for the second fermentation. Regarding the sensory analysis, important differences can be observed due to the type of inoculated yeast. Only a minor influence on the physical–chemical parameters was registered.

## 1. Introduction

Wine’s general quality, its stability, and organoleptic parameters depend on the physicochemical composition of raw materials, environmental conditions, and viticulture management. Nowadays, the alcohol concentration of wines has increased because of various agents, especially climate change [1,2]. At the same time, many consumers require lower alcohol products (9–13% *v/v*) as a consequence of health and social aspects, such as traffic restrictions [3,4].

Several techniques can be applied to produce low alcohol wines, mainly by using must with low levels of sugar concentrations, selected yeasts, or an earlier interruption of alcoholic fermentation [5]. To obtain a predetermined alcohol strength in wines, diverse practices (heat or membrane-based processes) can be employed. However, when using heat, a loss of important volatiles appears [6,7]. Several membrane-based procedures could be used to reduce the wine alcohol content [8], aiding in preserving the sensory characteristic of the initial wine [5]. These procedures (i.e., nanofiltration, reverse osmosis) have an important benefit: low energy consumption when working at decreased to moderate temperatures. Reverse osmosis represents a successfully employed procedure for reducing the alcohol strength and presents the benefit of generating insignificant negative impact on wine structure and composition (preserves aroma compounds and sensory features), since it is performed at low temperatures [9].

Sparkling wine production and consumption have constantly increased in the last decade and show no sign of slowing down. Consumption of this beverage shows a change from mainly festive to more regular occasions and a less specific manner [10]. In conformity with the traditional technique, these products undergo a double fermentation procedure, so after the first alcoholic fermentation, the wines are subjected to a second one by adding tirage liquor [11]. The aroma profile constitutes a major factor determining the sparkling wine’s typicity and quality but also its acceptability and competitiveness on the market. The traditional method usually generates a rich sensory profile [12] with over 800 different compounds that represent the volatile fraction of wine, but only a few of them are odor-active [13,14]. The volatile fraction comprises several classes of organic compounds in sparkling wines, such as esters, alcohols, organic acids, ketones, aldehydes, and terpenes [15]. Their concentration depends on the variety, meteorological, or biological aspects and wine-making practices [16,17]. The activity of yeasts strains during the alcoholic fermentation is regarded as a significant agent contributing to the volatile fraction and organoleptic feature of sparkling wines [18]. The aroma profile increases its complexity throughout the fermentation due to the synthesis of significant volatile compounds via *Saccharomyces cerevisiae* yeast strains and enhancement of varietal aroma precursors. The type and quantity of the synthesized volatile substances are dependent on multiple factors, for example the nitrogen concentration of the must, fermentation temperature conditions, and inoculated yeast strain [19].

Ethanol is predominant in wine, and it can modify the sensory perception of aroma compounds. Volatile compounds can be obtained from a variety of sources (raw material, fermentations, or aging stage) and have distinct physical–chemical properties, such as polarity, volatility, and odor impact as a result of the functional groups that exist in the molecule [20,21].

Yeasts present an important role defining sparkling wine’s features, including ethanol content, carbon dioxide overpressure, mannoproteins, and precursors of aroma compound levels. Most of the revealed composites manifest a positive contribution to the sensorial properties and foaming characteristics of the final product [22]. According to di Gianvito et al. [21], distinct flocculent *Saccharomyces cerevisiae* wine strains with diverse flocculation degrees can generate a substantial diversification of aroma molecules in terms of quantitative and qualitative views. Since the in-bottle fermentation of sparkling wines is usually conducted by few oenological products based on *Saccharomyces cerevisiae* strains [23], one could suggest the exploitation of the natural multiplication of the yeast population that aimed to produce variability in sparkling wines during the refermentation step. All yeast types can contribute to the formation of aromatic compounds through specific metabolic pathways. The results obtained at the end of the aging time (18 months) prove that important variances between the samples are obtained for the alcohol level, the achieved carbon dioxide pressure, and the sensorial traits of final samples.

Numerous studies have focused on the volatile fraction of sparkling wines and its sensorial implication [10,11,17,24]. Englezos et al. [25] followed the impact of some mixed fermentations of *Starmerella bacillaris* with different *Saccharomyces cerevisiae* strains on the volatile and physicochemical configuration of some wines obtained from Barbera varieties. Lower levels of ethyl alcohol, ethyl acetate, and acetic acid, increased amounts of higher alcohols, and pleasant smell esters register into the wines obtained with mixed cultures compared to those fermented only with *Saccharomyces cerevisiae* yeasts. Lencioni et al. [26] studied the evolution of volatile compounds during the alcoholic fermentation with mixtures of selected strains by *Zygotorulaspora florentina spp.* and *Saccharomyces cerevisiae* spp. compared with fermentation conducted only with *Saccharomyces cerevisiae* spp. Data showed a significant increase of 2-phenylethanol and a reduction of volatile acidity in the case of variants obtained with mixtures of yeasts.

Following the consumers’ tendency to prefer lower alcoholic beverages corroborated with the new generation’s wish for different organoleptic sensations, this article intends to observe if different specific commercial yeasts (randomly selected) manifest a significant impact on the volatile composition of experimental sparkling wines. The novelty of this study consists in comparing the impact of different yeast strains carrying out the secondary fermentation in sparkling wines production on the characteristics of the final product. In addition, the base-wine was obtained through reverse osmosis and had a predetermined alcoholic concentration, maintaining it at a lower level, as requested by the Z generation.

## 2. Materials and Methods

### 2.1. Grapes and Wine-Making Procedure

Five sparkling wine variants from the Muscat Ottonel grape variety were obtained. The grapes were manually harvested in autumn of 2018 at full maturity from Iași vineyard, Romania. The experimental wine (V0) had 12.5 % vol. and reverse osmosis was used for obtaining the base-wine (V0’), with a predetermined alcoholic concentration (10.5% vol.). 

The alcoholic fermentation was started by yeast strains inoculation (*Saccharomyces* spp.) at controlled temperature (18 °C). After the first fermentation, the tirage liquor (a mixture of selected yeast strains, 24 g L^−1^ sugar and wine) was added, and after that, the experimental mixture was bottled. The sugar concentration determines the sweetness degree of the wine and its pressure in the bottle. 

For the second fermentation, four commercial yeasts (IOC FIZZ™, IOC DIVINE™, LEVULIA CRISTAL™, IOC 18-2007™) were compared (resulting in V1, V2, V3, and V4 variants). The analyzed products are commonly used for sparkling wine production and recommended by the Institut Œnologique de Champagne. Each commercial product was inoculated according to the producer’s specification and legislation in force (20 g/hL). 

The second fermentation (that took place in the bottle) and aging in contact with lees (at 12 °C) lasted 15 months. After the aging phase, gravity drives the sediment lees to the bottle’s neck. The remuage process has been performed by manually rotating every bottle around 1/8 of a turn for 15 days. Bottle inclination is progressively modified until they are perpendicular on the rack. Disgorging was applied by freezing at −25 °C the upper part of the bottle, making it easier for the pressure built in the bottle to eliminate the lees. As the majority of the yeasts have either been expelled at the disgorgement stage, the sparkling wine in the bottle was then clear. After the disgorging phase, the expedition liquor was added, which is also known as “dosage” operation. In the end, the corks, labels, and muselets/wire cages were added. 

Samples were stored under controlled conditions (70% humidity, 8 °C temperature, and no light exposure) and analyzed after 6 months. The experimental samples were measured in triplicate, from three random bottles, three times each. 

### 2.2. Chemicals

A C7-C40 hydrocarbon mixture in hexane (Sigma-Aldrich, Cat. Number U-49451) was used for the determination of LRI in the HP-5MS, GC capillary column. All reagents and standards used were of analytical grade and supplied by Sigma-Aldrich or Merck.

### 2.3. Methods of Analysis

Physical–chemical parameters were performed according to the International Organization of Vine and Wine Compendium methods of analysis (2019): total (g L^−1^ tartaric acid) and volatile acidity (g L^−1^ acetic acid) by titrimetric methods, alcoholic strength (using a Dujardin-Salleron D.E. 2000 model for the simple distillation; % vol.), pH and density (using specific instruments), reductive sugars (g L^−1^) by Luff–Schoorl assay, free and total sulfur dioxide (mg L^−1^) by the iodometric method and non-reductive extract (g L^−1^) by Tabarié’s formula.

Volatile compounds were quantified using a GC-7890A chromatograph system, an MSD 5975 instrument purchased from Agilent Technologies, and a Multi-Purpose-Sampler from Gerstel (Germany), which were all governed via the software Chemstation (Agilent Technologies) and Maestro (Gerstel). The determination method of volatile compounds was managed according to the description of Vararu et al. [27] by a rapid stir bar sorptive extraction technique. A polydimethylsiloxane (Gerstel) Twister film was used (10 mm lengthy and 0.5 mm diameter). For the extraction, a 10 mL vial was filled with 0.5 mL experimental sample, and 0.1 mL of internal standard solution was prepared by adding 0.4464 mg L^−1^ ethyl nonanoate in ethyl alcohol (at high purity) and a solution of 12 % (*v/v*) ethyl alcohol adjusted to pH 3.5 using 2.6 g L^−1^ tartaric acid and 2.2 g L^-1^ potassium bitartrate to obtain the necessary volume (10 mL). After that phase, the Twister was introduced in the vial and homogenized at 20 °C, 1200 rpm, for 100 minutes. In that sense, a Variomag Multipoint 15 magnetic stirrer (Thermo Fisher Scientific, Inc.) was used. At the end, the Twister was removed, cleansed with distilled water, dried with cellulose material, and then transferred to a desorption tube into a Thermal Desorption Unit (Gerstel). The volatile constituents were thermally desorbed using an initial temperature of 35 °C for 0.1 seconds and 120 °C per minute ramp to 280 °C for 10 min and a helium stream at 16 mL/minutes in splitless mode into a Cooled Injection System (Agilent Technologies) equipped with an inlet liner packed with Tenax (3 × 2 mm). The cooled Injection system was preset at 25 °C temperature (for 0.05 seconds), at 12 °C per second ramp to 280 °C (for 7 minutes); helium inlet flow, 16 mL per minute. The gas-chromatograph system (7890A) was furnished with an HP-5MS fused silica capillary column (with 30 m length, 0.25 mm diameter, and 0.25 μm film thickness) from Agilent Technologies (USA). The oven initial temperature was fixed at 50 °C for 2 minutes and then increased with 4 °C per minute to a final temperature of 190 °C that was kept constant for 10 minutes. The mass selective detector was utilized in the electron impact mode, at 70 eV, using 35 to 550 Da range, at 150 °C temperature. The experimental samples were measured in triplicate, from three random bottles, three times each. Peak identification of the aroma components was done comparing the mass spectra results with data collection of Wiley7N and NIST08 libraries.

Regarding the sensory perception, a professional panel of 20 tasters (represented by winemakers, laboratory personnel, and researchers) evaluated the obtained experimental samples by defining the intensity (from 0—absence to 5—maximum) of some predetermined aroma descriptors. 

Statistical analysis was performed using XLSTAT software in Office Excel Package. The analysis of variance on volatile compounds was developed using the Anova one-way test. Since the Anova does not reveal which means are different from which, the Tukey’s honestly significant difference test was used to detect significant differences at *p* = 0.05 on 95 % confidence intervals. In addition, Principal Component Analysis describes the changes in the composition of volatile compounds of samples obtained with different yeasts. All results were presented as mean plus standard deviation.

## 3. Results and Discussion

### 3.1. Physical–Chemical Characteristics

The physical–chemical parameters of the analyzed sparkling wine samples are illustrated in Table 1. The type of inoculated yeasts showed only a minor impact on the physical–chemical parameters whose levels are within the limits allowed by International Organization of Vine and Wine (OIV) normatives [28]. Regarding the first two parameters (density and titratable acidity), significant differences can be observed between sparkling wine variants and the base wine used for its production. 

The experimental samples showed the highest total acidity in V2 and V3 (6.9 g L^−1^ tartaric acid), while the lowest value was registered in V4 variant (6.6 g L^−1^ tartaric acid). The pH presented similar results in all samples (3–3.1), with no important difference observed. 

The lowest production of SO_2_ was registered in V2 and V3 variants. Regarding the volatile acidity, the V2 sample displayed the highest value. These results showed a lower performance of fermentation conducted with the inoculated yeast cultures.

The amount of total sugars (0.7–3.4 g L^−1^) in V2 and V3 variants is more than double compared to V1 and V4. The alcoholic strength of sparkling wines varied from 11.3% vol. (V2 and V4) to 11.6% vol. (V1 and V3). 

Overall, all yeasts were able to complete fermentation (residual sugar content < 2 g L^−1^). 

Parameters such as density, total acidity, residual sugar, and non-reductive extract have dependent values, with important differences between samples. 

Comparable results were presented by Benucci and Esti [29].

### 3.2. Volatile Fraction

The quantified volatile compounds (represented by esters, acids, alcohols, and terpenes) were separated into their chemical classes. The identified substances and their correlated odor descriptors are presented in Table 2.

Esters contribute to sensory features of wines, being responsible for their floral and fruity notes. Their concentrations are dependent on various factors, including yeast species, temperature, and aeration degree during alcoholic fermentation and sugar content [30,31]. The majority of esters represent by-products of yeast metabolic action, with higher content in wine after cell division has slowed or essentially ceased. Straight-chain forms are synthesized from esterification of the corresponding acids, which have been activated by acyl-coenzyme A synthetases [32]. Esters such as ethyl octanoate, ethyl decanoate, ethyl laureate, isopropyl myristate, ethyl palmitate, and ethyl oleate were identified in analyzed samples. Their levels varied depending on the inoculated yeasts. According to Muñoz-Redondo et al. [10], several esters are considered markers of the second fermentation.

Isoamyl acetate is usually derived from yeast metabolism during the alcoholic fermentation. This compound contributes to a banana-like note and gives complexity to white wines [17,31]. Its concentrations varied from 11.71 μg L^−1^ in the V2 sample to 22.78 μg L^−1^ in the V4 sample. 2-Phenethyl acetate is generally produced by yeasts from phenolic precursors during the maturation stage and is characterized by sweet honey notes and flowers. The highest level of this compound was identified in the V1 and V3 samples, while the lowest concentration was registered in the V2 variant. According to Genovese et al. [33], ethyl decanoate (with floral odor properties) and 2-phenylethyl acetate (such as rose perfume) can present synergistic effect, even at reduced levels. The values of 2-phenylethyl acetate reported by Torchio et al. [34] were comparable with our results (22.33 to 47.72 µg L^−1^). 

Diethyl succinate is typically formed through alcoholic fermentation. The V2 sample displayed the highest level of this compound (62.58 μg L^−1^). According to de Souza Nascimento et al. [17], this compound was one of the most relevant esters in the volatile profile of Chenin Blanc sparkling wines. According to Torrens et al. [13] and Riu-Aumatell et al. [35], diethyl succinate represents one of the “aging esters” whose levels can show significant increases in contact with yeast cells through the second fermentation.

Acids can originate from the grape plant but also from the alcoholic fermentation, resulting in some fatty acids that have sensorial properties but can also supplement other roles. A combination of decanoic and octanoic acids is necessary to get a lasting inhibitory effect on yeast growth [35,36]. In the analyzed samples, the octanoic acid content varied from 580.64 μg L^−1^ in the V1 variant to 258.79 μg L^−1^ in the V2 sample. Decanoic acid reached a maximum concentration in the V1 sample (145.25 μg L^−1^) and a minimum in the V2 sample (11.36 μg L^−1^).

Alcohols represent secondary aromatic components derived from sugars and amino-acids transformation during the fermentation process, with a significant influence on wine’s sensorial profile [17,31]. Concerning the alcohols level, isoamyl alcohol, 4-octanol, 1-heptanol, and 2-phenylethyl were the most representative in resulting samples. Isoamyl alcohol generally accounts for more than 50% of all fusel alcohols fractions [37]. The experimental samples ranged from 1019.50 μg L^−1^ in the V3 sample to 485.91 μg L^−1^ in the V2 sample. These compounds were also identified in high proportion in Muscat Ottonel wines by Călugăr et al. [38].

A small part of the fusel alcohols may originate from grape-derived aldehydes by the reductive denitrification of amino acids or throughout the synthesis of sugars [39,40]. The appearance of higher alcohols through the fermentation stage is usually influenced by the wine-making techniques, inoculated yeasts, low amino-acids levels, low temperature, and reduced pH degree [40,41]. The amount of higher alcohols produced during fermentation of the grape juice significantly varied according to the inoculated yeast. 1-heptanol was identified in large quantities in the V2 variant, assuring a pleasant vegetal odor and fruity notes (apples and banana). Phenylethyl alcohol, a volatile compound with pleasantly sweet, floral, and honey odors was detected in all analyzed samples. Larger quantities of this compound were identified in V1 (1150.12 μg L^−1^) and V4 (683.46 μg L^−1^) samples. Its presence in wine is probably due to the degradation of amino acids, as it is shown in the Ehrlich pathway. The production of phenylethyl alcohol depends on the temperature level and inoculated yeast strains [42]. Data published by Torrens et al. [13] and Jaganatić Korenica et al. [43] also showed high proportions of phenylethyl alcohol.

Terpenes represent secondary metabolites that originate from the grapes. However, the biosynthesis of monoterpenes by *Saccharomyces cerevisiae* in the absence of grape-derived precursors was indicated to be a possible origin for aroma compounds in wine [44]. Terpenes play an important role in defining the floral odor of wines that reminds of roses and are usually specific for the Muscat de Alexandria and white Frontignac grapes [45]. L-Linalool gives a fresh floral aroma to wines, which is reminiscent of spices and lemon notes. The highest concentration of linalool is usually registered in aromatic and semi-aromatic varieties, such as Muscat Ottonel, Tămâioasă Românească, Sauvignon blanc, and Fetească albă [46]. The V1 variant showed the highest level in L-linalool (138.86 μg L^−1^), followed by V3 (120.43 μg L^−1^), V4 (44.31 μg L^−1^), and V2 (16.65 μg L^−1^). This compound is converted by the action of acids into geraniol, nerol, and α-terpineol, respectively [45]. α-terpineol usually gives wine a fruity (melon) odor and floral (lilac) perfume. It is formed out of monoterpene–glycosides in an acid medium [47]. The highest concentration was identified in the V1 sample (42.79 μg L^−1^), followed by V3 (41.40 μg L^−1^), V2 (28.19 μg L^−1^), and V4 variants (24.19 μg L^−1^). This compound can originate from the grape (in low concentrations) and have a high olfactory perception value [45]. High levels of linalool and α-terpineol were also identified in Moscato Giallo wines by Marcon et al. [48]. Comparable concentrations of linalool were identified in Muscat de Alexandria wines by Lanaridis et al. [49].

A significant impact of supplemented yeasts on the volatile profile was observed. V1 and V3 variants have been remarked to have the highest influence on the majority of aroma compounds. The null hypothesis that the type of yeast did not affect the concentrations of the volatile compounds was rejected, and the alternative one that in fact the yeast did affect the volatile content of the analyzed sparkling wines was confirmed (*p* < 0.05). Regarding the results of Tukey’s HSD (honest significant differences) test (Table 3), a significant difference between V1 and V3 in the case of 1-heptanol and α-terpineol variances can be observed. In addition, statistically significant differences between V2 and V4 samples on the butyric acid, linalool, and α-terpineol concentrations were registered. In the case of diethyl succinate and decanoic acid, the significant difference was represented by the V3 and V4 groups (*p* < 0.05).

The PCA test describes the variations of the composition of volatile compounds of sparkling wines produced by different commercial yeast strains. Table 4 presents the loadings for each variable on the selected factor as well as the eigenvalue and the cumulative variance. The variables marked with bold have the major contribution to the explanatory meaning of the three factors. The first factor described 59.82% of the data variability and was strongly correlated with most of the identified volatile compounds (ethyl octanoate and decanoate, 2-phenethyl acetate, ethyl laurate and laurate, hexanoic, octanoic, decanoic and 9-decenoic acid, isoamyl alcohol, 4-octanol, phenylethyl alcohol, linalool L, and α-terpineol). Therefore, these components are highly correlated with most of the volatile compounds identified in analyzed samples.

**Figure 1:**
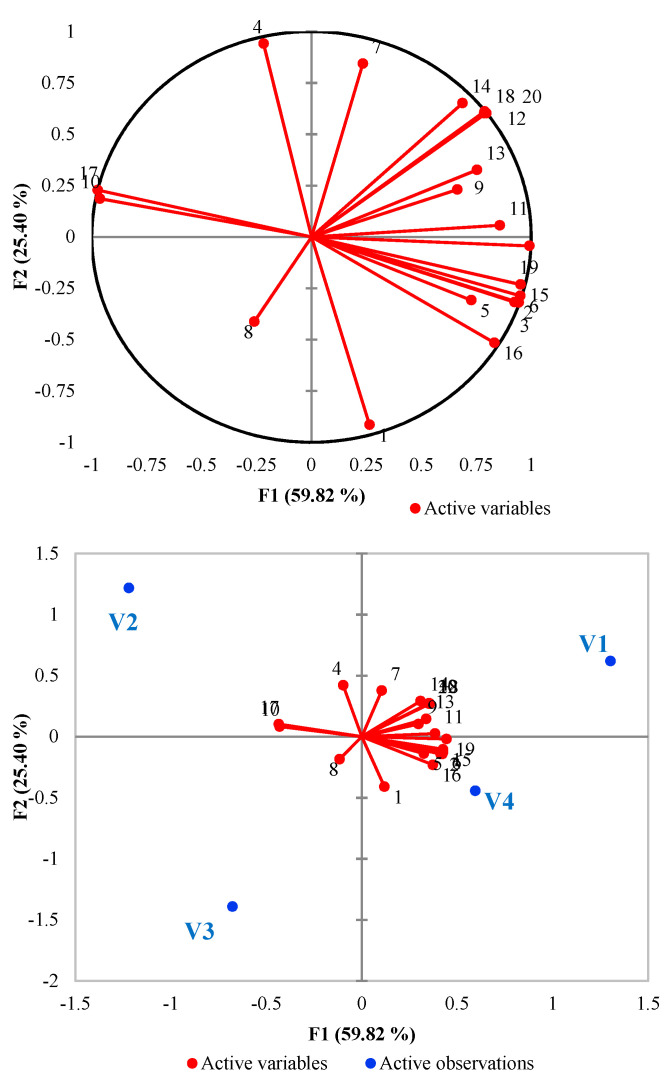
Principal component analysis based on the gas-chromatograph results: **1**, Isoamyl acetate; **2**, Ethyl octanoate; **3**, Ethyl decanoate; **4**, Diethyl succinate; **5**, 2-Phenethyl acetate; **6**, Ethyl laurate; **7**, Isopropyl myristate; **8**, Ethyl palmitate; **9**, Ethyl oleate; **10**, Butyric acid; **11**, Hexanoic acid; **12**, Octanoic acid; **13**, Decanoic acid; **14**, 9-Decenoic acid; **15**, Isoamyl alcohol; **16**, 4-Octanol; **17**, 1-Heptanol; **18**, Phenylethyl alcohol; **19**, Linalool L; **20**, α-terpineol; V1—FIZZ^™^, V2—IOC DIVINE^™^, V3—LEVULIA CRISTAL^™^, V4—IOC 18-2007^™.^

Figure 1 encompasses the first two principal components, which explain around 85% of the total data variability. The first principal component that explained most of the total variability of the data (59.82%) was strongly correlated with isoamyl acetate, ethyl decanoate, ethyl laurate, isoamyl alcohol, and linaool showed in all cases factor loadings greater than 0.90. For the second principal component, diethyl succinate and isopropyl myristate showed high and positive values. 

The correlation circles (Figure 1) show a projection of the initial variables in the factors space. It can be observed that 1-heptanol is positively correlated with butyric acid (r close to +1) but negatively correlated with isopropyl myristate (r close to −1). In addition, linalool and ethyl decanoate are positively correlated, while linalool and butyric acid show a negative correlation.

Ehyl octanoate, ethyl decanoate, and isoamyl alcohol, the most predominant volatile substances in the analyzed samples, are also positively correlated. On the other hand, they are significantly negatively correlated with butyric acid and 1-heptanol (on the opposite side). The biplot chart enables observations and variables to be made on a two-dimensional map and identification of the trends. As can be seen in the mentioned figure, the variables related to factor 1 permit differentiating the samples by volatile fraction.

Since the purpose was to evaluate the influence of inoculated yeasts on the volatile fraction of experimental sparkling wines, the data confirm that different yeast can generate different levels of volatile compounds. 

**Figure 2:**
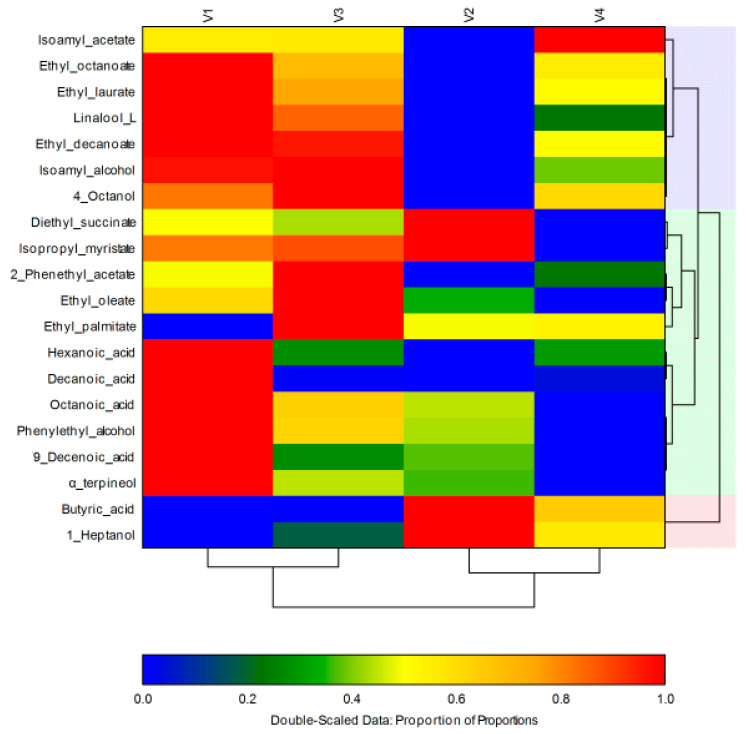
Heat map obtained using the concentration of each volatile compound in resulted experimental sparkling wines. Clusters linked to the grouping of volatiles and samples were designed. Samples represented with blue color showed the lowest concentrations of the separated compound, while the highest levels are represented in red.

The heat map (Figure 2) was obtained using the identified concentration of each volatile compound according to Table 2, expressing a visual assessment of the correspondences (similarities and differences) concerning the volatile fraction of samples. Data are exposed in a grid where each row signifies a quantified volatile compound and every column represents a sample. The color of the obtained boxes and its intensity is used to represent changes on each compound concentration. In the figure, it can be observed that red color indicates the highest concentrations of each substance and blue represents the lowest. The order of the rows is determined by performing hierarchical cluster analyses of the rows. The first variant was noted for higher concentrations for most of the identified volatile compounds, followed by V3. This means that the inoculated yeasts have been shown to be much more effective in enriching it with flavor compounds.

Several works studying similar products [17,21,53,54,55] have reported significant influence in sparkling wine’s aroma compounds and only a minor influence on the physical–chemical parameters.

Many studies that refer to the volatile compounds evolution throughout the aging of sparkling wines presented opposite results due to different experimental circumstances and the simultaneous degradation and synthesis of volatile fraction that occurs through the aging stage of wine with yeast. It results that at any given time, either of these processes can predominate. According to Torrens et al. [51], different commercial yeast strains present a significant impact on the chemical and volatile composition of the sparkling wines, with major repercussion on their sensory profile.

## 4. Sensory Characteristics

The sensory perception of sparkling wines is given by the interaction of different volatile constituents. The character of sparkling wine is usually influenced by its effervescence, sweetness, acidity, or bitterness and is generated by non-volatile compounds that are soluble in water or an alcohol mixture [56].

**Figure 3:**
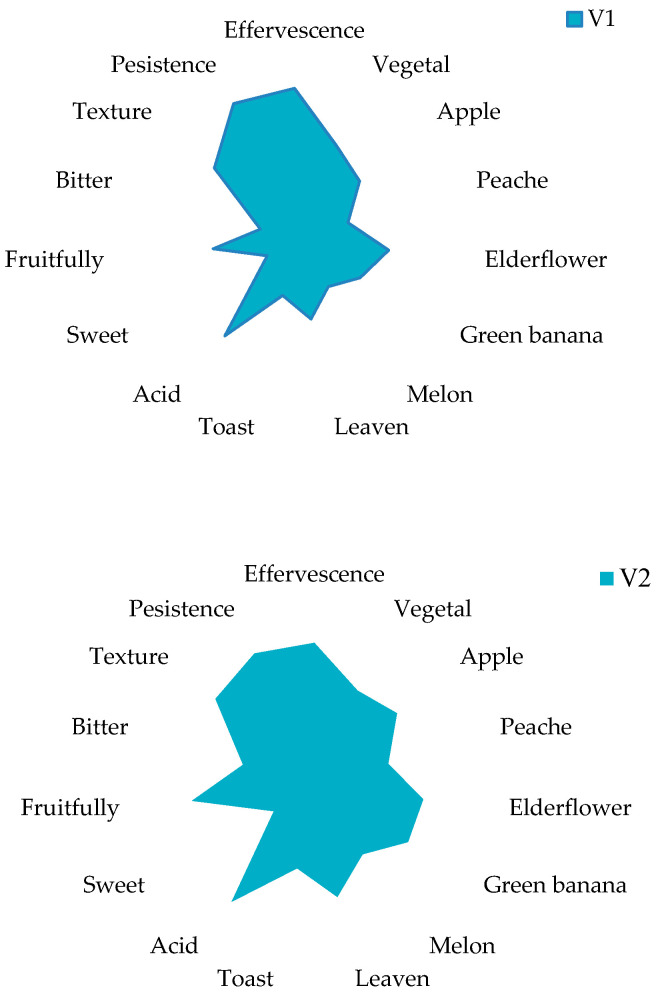

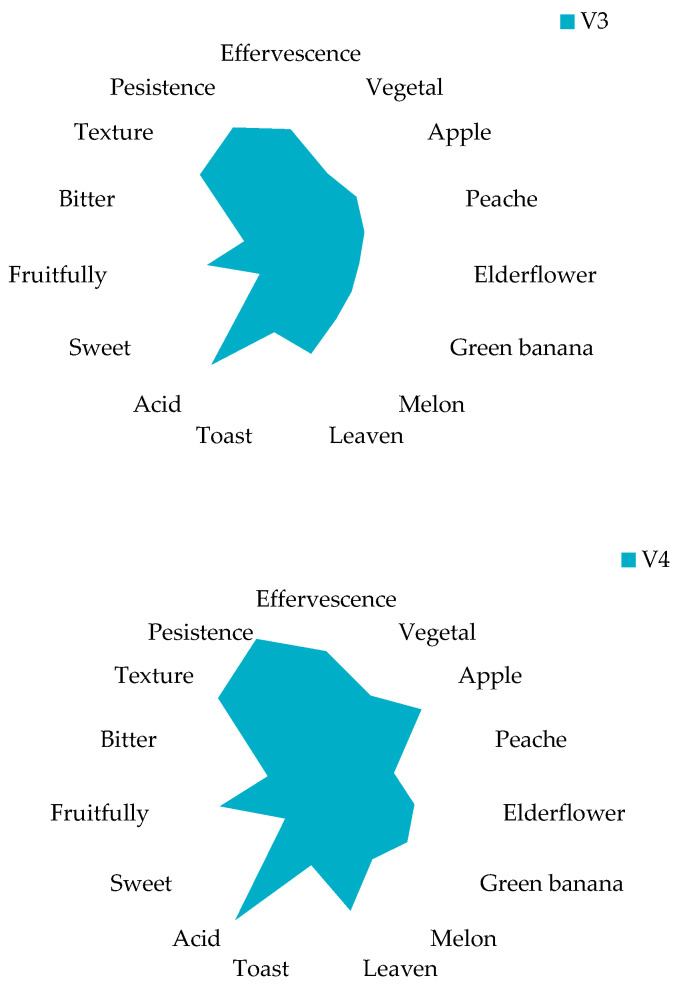
Sensory perception of experimental sparkling wines. The odor intensity of the analyzed parameters was evaluated by means of a hedonistic scale starting with 0—absence to 5—maximum.

According to the sensory analysis (Figure 3), major differences can be observed due to the type of inoculated yeasts. All sparkling wines were characterized as balanced, with great persistence, acidity (that imprinted freshness), and good texture (especially V4 sample). The V1 variant was remarked for its floral odor (elderflowers) while fruity notes were dominant in the V2 sample (especially green banana).

Regarding the odor descriptors (Table 2), the resulting sparkling wines are defined by their fruity (especially banana-like and apple) and floral notes (elderflower), due to their high levels of esters (e.g., ethyl octanoate and ethyl decanoate). Isoamyl acetate, ethyl palmitate, 4-octanol, or 1-heptanol did not significantly contribute to the final aroma profile of experimental samples.

Regarding the correlation between the volatile compounds and sensorial perception, the fruity notes (apple, green banana, peach) of experimental samples can be explained by the presence of ethyl octanoate, ethyl decanoate, or diethyl succinate. The floral odor is mainly due to the high concentrations of phenylethyl alcohol.

**Figure 4:**
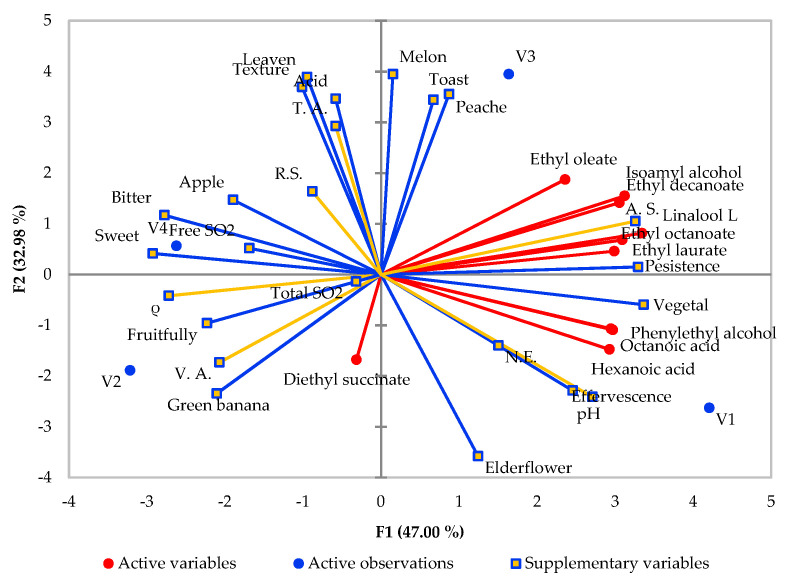
Principal component analysis based on the sensory analysis results, predominating volatile compounds, and wine basic parameters.

In Figure 4, F1 creates a visible separation of the samples regarding the aroma compounds concentration and their odor intensities. For this plot, ten compounds with higher levels were chosen. Samples that were appreciated to have higher levels of identified compounds and more intense descriptors are positioned on the right of the plot (V1 and V3), while the samples with lower intensities are situated on the left of the plot. V2 and V4 variants presented similar odor intensities. 

## 5. Conclusions

According to the results, yeasts can influence the final quality of wines in varying degrees. Considering the physicochemical characteristics, the type of inoculated yeasts showed a minor but important impact on the physical–chemical parameters. Parameters such as density, total acidity, residual sugar, and non-reductive extract have dependent values, with important differences between samples. Data showed a significant contribution of commercial selected yeasts to the enrichment of the volatile fraction of wines. Regarding the sensory characteristics, key differences can be observed due to the type of inoculated yeast. Ethyl octanoate and ethyl decanoate were representatives for all variants, defining their fruity (especially banana, apple) and floral notes (elderflower). V1 and V3 variants show the highest concentrations of the majority of aroma compounds while V2 and V4 presented the lowest levels. This means that the inoculated yeasts have been shown to be much more effective in enriching with flavor compounds. These results can contribute to the optimization of wine-making technology for obtaining low alcohol products with rich aroma profile. 

## Figures and Tables

**Table 1:** Physical–chemical parameters of obtained samples.

Samples	ρ	T. A. (g tartaric acid L^−1^)	V. A. (g acetic acid L^−1^)	A. S. (% vol.)	Free SO_2_ (mg L^−1^)	Total SO_2_ (mg L^−1^)	R. S. (g L^−1^)	N. E. (g L^−1^)	pH
**V0**	0.9932 ± 0.0001	6.3 ± 0.07	0.30 ± 0.02	12.5 ± 0.03	18 ± 0.47	72 ± 0.47	3.4 ± 0.13	17.0 ± 0.14	2.9 ± 0.02
**V0’**	0.9921 ± 0.0001	6.2 ± 0.03	0.30 ± 0.01	10.5 ± 0.05	17 ± 0.47	71 ± 0.00	3.2 ± 0.05	15.2 ± 0.13	2.8 ± 0.05
**V1**	0.9905 ± 0.0003	6.7 ± 0.02	0.30 ± 0.01	11.6 ± 0.07	5 ± 0.00	56 ± 0.47	0.7 ± 0.02	14.5 ± 0.04	3.1 ± 0.01
**V2**	0.9908 ± 0.0001	6.9 ± 0.01	0.35 ± 0.01	11.3 ± 0.00	5 ± 0.00	49 ± 0.47	1.9 ± 0.01	13.3 ± 0.05	3.0 ± 0.01
**V3**	0.9906 ± 0.0001	6.9 ± 0.03	0.30 ± 0.05	11.6 ± 0.00	5 ± 0.47	51 ± 0.47	1.9 ± 0.01	13.5 ± 0.01	3.0 ± 0.01
**V4**	0.9907 ± 0.0002	6.6 ± 0.01	0.30 ± 0.09	11.3 ± 0.07	8 ± 0.47	64 ± 0.00	0.7 ± 0.02	14.3 ± 0.01	3.0 ± 0.01
***p*-value**	0.0001	0.0001	0.0772	0.0772	0.0772	0.0772	0.0772	0.0772	0.0772

ρ—density; T.A.—total acidity; V.A.—volatile acidity; A.S.—alcohol strength; R.S.—residual sugar; N.E.—non-reductive extract. The values are presented as mean and standard deviation of three experimental bottles (triplicate). Analysis of variance was carried out by comparing each wine variant with the base wine used for its production.

**Table 2:** Volatile fraction of resulted sparkling wine.

No	V. C. (μg L^−1^)	V1	V2	V3	V4	Odor Descriptors	References
**ESTERS**							
1	Isoamyl acetate	17.83 ± 0.06 *	11.71 ± 0.15 *	17.89 ± 0.23 *	22.78 ± 0.11 *	fruity, banana	[20]
2	Ethyl octanoate	7998.72 ± 0.15 *	5285.90 ± 0.08 *	7162.47 ± 0.21 *	6789.59 ± 0.31 *	fruity, banana, apple, pineapple, pears, floral, sweet, soap	[7,50]
3	Ethyl decanoate	2177.35 ± 0.35 *	985.37 ± 0.20 *	2126.20 ± 0.11 *	1593.61 ± 0.30 *	fruity, apple, waxy, oily	[50]
4	Diethyl succinate	53.58 ± 0.90 *	62.58 ± 0.01 *	49.52 ± 0.11 *	54.40 ± 0.57 *	fruity, floral, waxy, dusty	[7]
5	2-Phenethyl acetate	34.87 ± 1.57 *	22.33 ± 0.81 *	47.72 ± 0.44 *	28.18 ± 0.16 *	floral, sweet, fruity, honey	[13]
6	Ethyl laurate	162.34 ± 0.51 *	56.71 ± 0.01 *	136.25 ± 0.45 *	110.49 ± 0.82 *	floral, fruity, grassy, woody	[27,50]
7	Isopropyl myristate	14.87 ± 0.17 *	16.17 ± 0.50 *	15.99 ± 0.11 *	13.98 ± 0.76 *	faint, oily, fatty	[27]
8	Ethyl palmitate	15.64 ± 0.98 *	7.89 ± 0.15 *	15.98 ± 0.15 *	8.51 ± 0.22 *	waxy, fruity, creamy and milky with a vanilla balsamic nuance	[13]
9	Ethyl oleate	159.21 ± 0.08 *	132.12 ± 0.16 *	198.97 ± 0.19 *	108.42 ± 0.23 *	fatty, oily, dairy, milky, waxy, tallow	[13]
**ACIDS**							
10	Butyric acid	nd	9.81 ± 0.57 *	nd	6.32 ± 0.11 *	cheese, rancid, sweet, animal	[7,50]
11	Hexanoic acid	326.09 ± 0.25 *	189.98 ± 0.11 *	227.50 ± 0.70 *	230.34 ± 0.45 *	fatty	[13,51]
12	Octanoic acid	580.64 ± 3.22 *	258.79 ± 2.23 *	367.50 ± 0.40 *	nd	cheese	[39]
13	Decanoic acid	145.25 ± 0.59 *	11.36 ± 0.06	13.01 ± 0.06 *	16.91 ± 0.14 *	rancid, sour, oily, unpleasant, woody	[50,52]
14	9-Decenoic acid	6.90 ± 2.25	6.75 ± 0.98 *	4.37 ± 1.30	4.00 ± 2.20	waxy orange, reminiscent of kiwifruit, fruity and milky, melon note	[27]
**ALCOHOLS**							
15	Isoamyl alcohol	1001.47 ± 0.23 *	485.91 ± 0.16 *	1019.50 ± 0.02 *	693.63 ± 0.50 *	alcohol, nail polish, bananas	[16,50]
16	4-Octanol	5.62 ± 0.59	5.53+0.75	6.13 ± 0.40	5.14 ± 0.56	-	-
17	1-Heptanol	5.89 ± 0.54 *	28.70 ± 0.04 *	10.06 ± 0.40 *	18.76 ± 0.45 *	musty, violet, herbal, woody, peony	[50]
18	Phenylethyl alcohol	1150.12 ± 0.23 *	884.56 ± 0.14 *	973.18 ± 0.03 *	683.46 ± 0.01 *	floral, rose, dried rose	[50]
**TERPENIC COMPOUNDS**							
19	Linalool L	138.86 ± 0.06 *	16.65 ± 0.55 *	120.43 ± 0.01 *	44.31 ± 2.22 *	citrus, floral, bois de rose, green blueberry	[50]
20	α-terpineol	42.79 ± 0.40 *	28.19 ± 0.14 *	41.40 ± 0.02 *	24.19 ± 0.85 *	pine like, lilac, citrus, woody, floral	[27]

The results are presented as mean plus standard deviation of three experimental sparkling wine bottles; V.C.—volatile compounds; n.d.—not detected; * statistically significant.

**Table 3:** Significant results of Tukey’s HSD post-hoc test.

Variables	Groups	Diff	*p*	95% Confidence Interval for Mean	
				Lower Bond	Upper Bond
Diethyl succinate	V3-V4	4.8800	0.0000	3.7558	6.0042
Butyric acid	V2-V4	−3.4900	0.0000	−4.0993	−2.8807
Decanoic acid	V2-V3	1.6500	0.0000	1.0073	2.2927
	V3-V4	3.9000	0.0021	3.2573	4.5427
1-Heptanol	V1-V3	4.1700	0.0000	3.3200	5.0200
Linalool L	V2-V3	−4.220	0.0011	−6.6216	−1.8184
α-terpineol	V1-V3	−1.3900	0.0065	−2.3872	−0.3928
	V2-V4	−4.0000	0.0000	−4.9972	−3.0028

**Table 4:** Factor loadings of the experimental samples.

	Factor 1	Factor 2	Factor 3
Eigenvalue	11.964	5.081	2.955
Variability (%)	59.820	25.404	14.775
Cumulative %	59.820	85.225	100.000
Isoamyl acetate	0.264	−0.915	−0.305
Ethyl octanoate	**0.924**	−0.317	−0.211
Ethyl decanoate	**0.944**	−0.319	0.089
Diethyl succinate	−0.219	**0.941**	0.257
2-Phenethyl acetate	**0.727**	−0.308	0.613
Ethyl laurate	**0.951**	−0.287	−0.118
Isopropyl myristate	0.234	**0.844**	0.483
Ethyl palmitate	−0.261	−0.413	**0.873**
Ethyl oleate	0.664	0.231	**0.711**
Butyric acid	−0.964	0.186	−0.192
Hexanoic acid	**0.857**	0.057	−0.512
Octanoic acid	**0.788**	0.611	0.075
Decanoic acid	**0.753**	0.326	−0.571
9-Decenoic acid	**0.687**	0.651	−0.321
Isoamyl alcohol	**0.952**	−0.232	0.202
4-Octanol	**0.832**	−0.516	0.203
1-Heptanol	−0.973	0.228	0.012
Phenylethyl alcohol	**0.796**	0.603	0.060
Linalool L	**0.992**	−0.044	0.117
α-terpineol	**0.786**	0.602	−0.139

The bold numbers indicate the higher weight of each compound in each factor.

## Data Availability

Data available in a publicly accessible repository.
